# On the Similarity of Functional Connectivity between Neurons Estimated across Timescales

**DOI:** 10.1371/journal.pone.0009206

**Published:** 2010-02-18

**Authors:** Ian H. Stevenson, Konrad P. Körding

**Affiliations:** 1 Department of Physical Medicine and Rehabilitation, Rehabilitation Institute of Chicago, Chicago, Illinois, United States of America; 2 Department of Physiology, Feinberg School of Medicine, Northwestern University, Chicago, Illinois, United States of America; The University of Western Ontario, Canada

## Abstract

A central objective in neuroscience is to understand how neurons interact. Such functional interactions have been estimated using signals recorded with different techniques and, consequently, different temporal resolutions. For example, spike data often have sub-millisecond resolution while some imaging techniques may have a resolution of many seconds. Here we use multi-electrode spike recordings to ask how similar functional connectivity inferred from slower timescale signals is to the one inferred from fast timescale signals. We find that functional connectivity is relatively robust to low-pass filtering—dropping by about 10% when low pass filtering at 10 hz and about 50% when low pass filtering down to about 1 Hz—and that estimates are robust to high levels of additive noise. Moreover, there is a weak correlation for physiological filters such as hemodynamic or Ca^2+^ impulse responses and filters based on local field potentials. We address the origin of these correlations using simulation techniques and find evidence that the similarity between functional connectivity estimated across timescales is due to processes that do not depend on fast pair-wise interactions alone. Rather, it appears that connectivity on multiple timescales or common-input related to stimuli or movement drives the observed correlations. Despite this qualification, our results suggest that techniques with intermediate temporal resolution may yield good estimates of the functional connections between individual neurons.

## Introduction

In the past few decades a number of methods have become available for estimating the interactions or functional connections between neurons or brain areas from neural signals [1], [2]. These techniques are beginning to shed light on how the brain is functionally organized [3], [4] and how populations of neurons process and encode information [5]. One of the advantages of this general approach is that estimates of functional connectivity can be made using signals from a number of different recording techniques from extra-cellular unit recordings [6], [7] and calcium imaging [8] to local field potentials [9] and fMRI [10]. Each technique provides information about network structure and dynamics on a different spatiotemporal scale. To be able to combine results from different recording techniques into a global understanding of interactions in the nervous system we need to know the relationship between functional connectivity estimated from methods with distinct timescales.

Functional connectivity analyses differ from anatomical connectivity in several important ways. While these analyses complement anatomy-based approaches for assessing connectivity such as wire-tracing [11]–[13], anti-dromic stimulation [14], or diffusion imaging [3], [15], their interpretation is typically more elusive. Unless one records with perfect temporal resolution from all neurons in the brain there will always be missing information. Unrecorded neurons, for example, may induce apparent functional connectivity between recorded neurons. Thus, an estimated network must be interpreted as an abstraction of the true network and true interactions [2]. Secondly, whereas wire-tracing and diffusion imaging provide information about stable anatomical connections (albeit on different spatial scales), the signals used to estimate functional connectivity generally differ in terms of biological origin (e.g. dendritic potentials or spiking activity) and spatiotemporal resolution. Functional connectivity estimated from a single recording technique can be useful for decoding external signals [5], [7] and understanding the structure of interactions at that scale, but building a complete picture of functional connectivity on multiple spatial and temporal scales may prove more difficult.

We may hope that functional connectivity calculated from different signals is similar because there are correlations between neural activity measured using different techniques. Various studies have shown that, at least in some cases, LFP and fMRI signals are well correlated with spikes [16], [17]. However, the relation between different types of signals is not always simple or consistent [18]–[22]. One possibility is that, even if the relationship between recording techniques is not entirely clear, network activity recorded using two different techniques may provide common information about interactions between neurons or areas of the brain. As such, it seems important to ask how much functional connectivity is affected by the resolution of recorded signals. Understanding the relationship between temporal resolution and inferred functional connectivity is also important as it may tell us how fast we should record data to enable efficient estimation of functional connectivity.

Here we compare functional connectivity estimates from multi-electrode spike data with spike data that has been altered to remove certain timescales or mimic known recording techniques. We find that functional connectivity estimated from these simulated recording techniques (fMRI, LFP, and Ca^+2^ recordings) and functional connectivity estimated from low passed signals matches the fast timescale connectivity fairly well for timescales down to ∼1 Hz. These results suggest that filtered signals may be used to efficiently estimate fast timescale functional connectivity. Finally, to test one possible origin for the observed slow timescale functional connectivity we use several spike simulations. One possibility is that slow interactions appear as a side-effect of fast (synaptic-like) interactions. To test this idea, we fit a spiking model to the original multi-electrode data and simulate from this model using fast timescales only. We find that the slow timescale functional connectivity estimated from these simulations is only very weakly correlated with the estimated fast timescale connectivity. The correlations between functional connectivity on different timescales are reduced, even when we attempt to include slowly varying external covariates (e.g. end-point velocity during reaching movements). The patterns observed in real data thus appear to be best explained by neuronal interactions on multiple timescales or unobserved common input.

## Results

### Temporal Filtering Analysis

**Figure 1:**
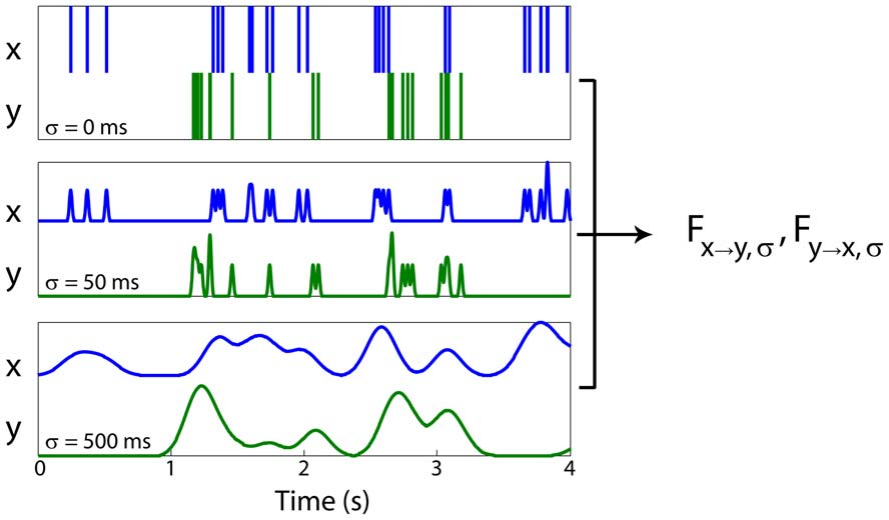
Filtering analysis. The Granger causality between each pair of neural signals (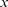 and 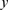) is calculated at different levels of smoothing (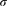) and down-sampling. This provides measures of functional connectivity from 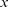 to 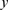 (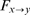) and from 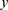 to 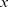 (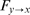) for each timescale. By comparing these measures across timescales we can examine how robust functional connectivity is to temporal filtering.

We analyze multi-electrode, single unit, spike data recorded from the motor cortices of two macaque monkeys (*Macaca mulatta*). After filtering and down-sampling the spike signals, we compute a measure of functional connectivity, the pair-wise Granger causalities from each pair of channels [23] (Fig. 1). Granger causality provides a metric for how much one signal improves prediction of another. It specifically measures the improvement given by adding a second signal to an auto-regressive linear model. Granger causality has been used to estimate interactions with a variety of signals, and here it provides an estimate of the strength of functional connectivity between neurons. Our goal is to compare functional connectivity estimated from different signal types. As a first step, we compare Granger causality estimated from the highest frequency spike signals with Granger causality calculated from filtered spike signals. Using this strategy we can examine how functional connectivity estimated from slow timescale signals relates to fast timescale connectivity. Correlations between these two functional connectivity estimates imply that functional connectivity calculated from filtered signals is predictive of functional connectivity at fast timescales.

**Figure 2:**
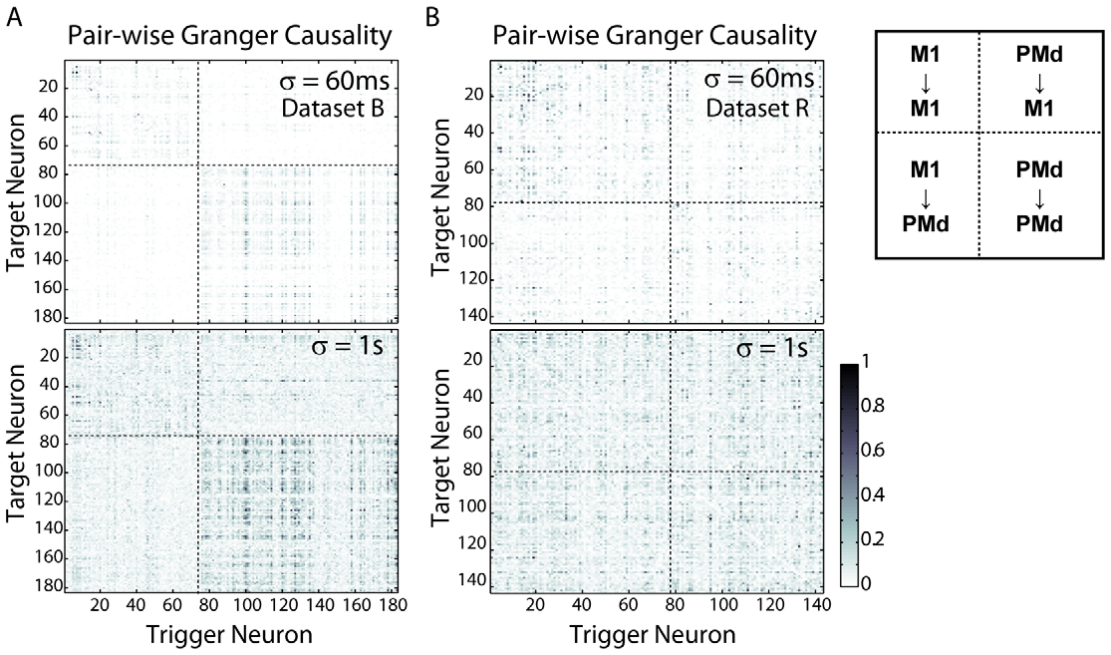
Results from two sets of multi-electrode spike data. (A–B) show matrices of pair-wise Granger causality (scaled to [0,1]) between each set of neurons at two levels of smoothing and down-sampling. The structure is consistent across a wide range of timescales. Note that neurons in M1 and PMd, separated by the dotted lines, tend to cluster together (especially in Dataset B).

**Figure 3:**
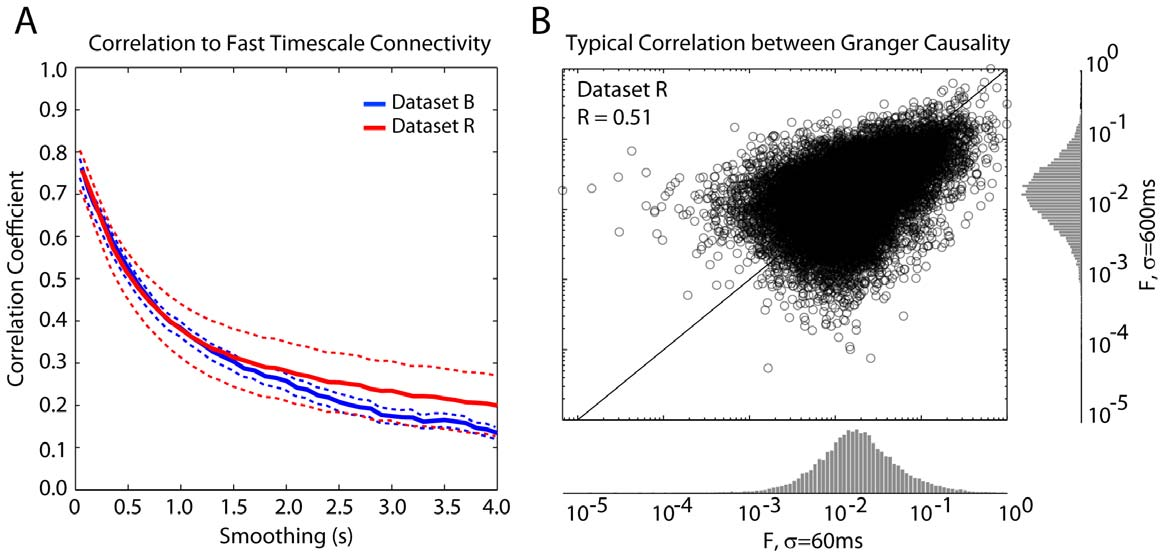
The correlation between functional connectivity across timescales is robust for all datasets down to ∼0.25 Hz. Correlation coefficients are calculated across folds (10 min segments). Error-bars denote standard error (SEM) across segments (N = 5).

We examine the full population of neurons from each of our datasets (143 for monkey R, 183 for monkey B) and divide the data into non-overlapping blocks of 10 minute duration. We then compute correlation coefficients between functional connectivity (Granger causality) estimated from different segments at varying levels of low-pass filtering (Fig. 2A and B). Cross-validation ensures that model comparisons are relevant and not due to over-fitting. We find that connectivity estimates are fairly robust to temporal filtering (Fig. 3). For instance, functional connectivity estimated after low-pass filtering at 1 Hz (Gaussian filter, σ = 1 s) is still significantly correlated with the fast timescale functional connectivity (R = 0.4). Moreover, the rate at which this correlation decays as a function of temporal resolution is conserved across animals and tasks. Dataset R was recorded while the animal performed center-out reaches, and dataset B was recorded while the animal was sleeping (non-REM, slow-wave sleep). This invariance suggests that, while functional connectivity itself is task dependent, there may be consistent relationships between functional connectivity across different timescales.

Qualitatively, the functional connectivity estimated from dataset B (sleep) has much more structure than that estimated from dataset R (reaching). Neurons in areas M1 and PMd tend to cluster together more closely (i.e. they have similar in- and outgoing connectivity) in dataset B than in dataset R. However, the distribution of directed Granger Causality in both datasets is in good approximation exponential, and the connectivity matrices in both cases are fairly symmetric. For any given pair of neurons, the connection in one direction (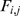) differs from the connection in the other direction (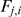), on average by 1.7% for dataset B and 1.6% for dataset R.

**Figure 4:**
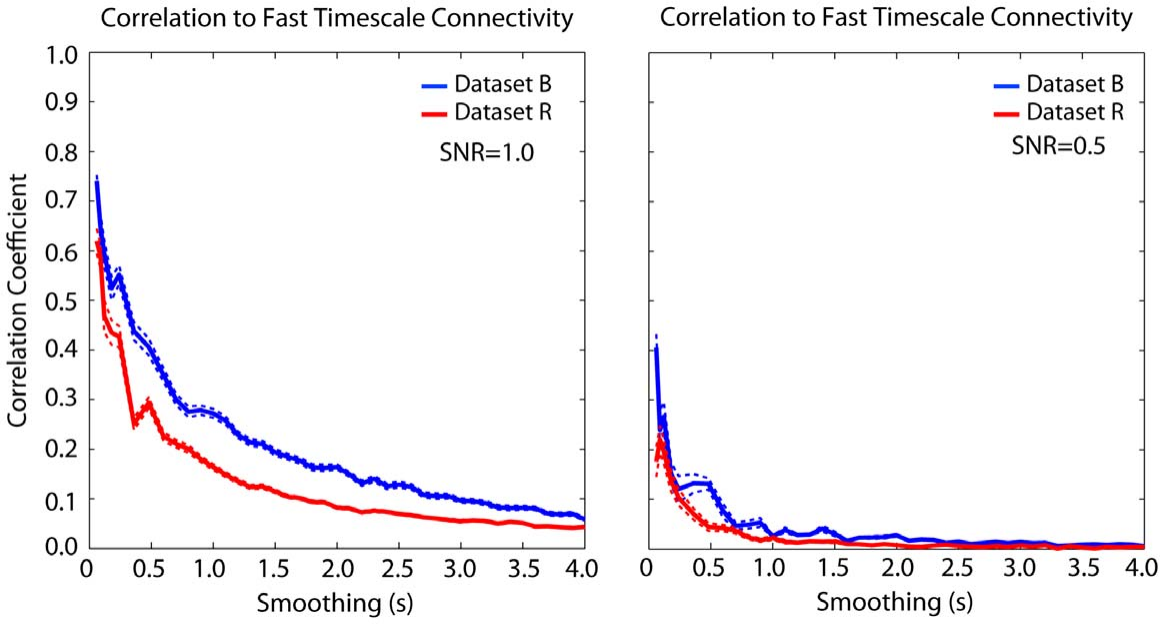
Effects of additive Gaussian noise. Adding uncorrelated Gaussian noise (SNR = 1) reduces the correlation slightly, but there is still substantial correlation at 1 Hz. Error-bars denote SEM across segments (N = 5).

Since the filtering and down-sampling procedure is noise-free, the correlations above may not reflect the true relationship between connectivity estimates that would be observed using two different (noisy) recording techniques with two different temporal resolutions. However, the correlation between fast and slow timescale connectivity estimates is fairly robust to additive, uncorrelated Gaussian noise. Connectivity between fast and slow (1 Hz) connectivity is well correlated for signal-to-noise ratios as low as ∼1 (Fig. 4, left). These results suggest that, at least to some extent, connectivity inferred from low-pass signals, with noisy, limited data, is predictive of fast timescale connectivity inferred from spikes. For comparison we show the correlation between connectivity at each timescale for a signal-to-noise ratio of 0.5 (Fig. 4, right). In this case, the correlations are much reduced.

It is important to note that the correlations are computed only between different segments of data (cross-validated). That is, we do not compare connectivity estimated from the exact same signal segments at different timescales. The reported correlations effectively lower-bound the relationship between connectivity at different timescales, since non-stationarity in the functional connections should only reduce correlations.

### Temporal Filtering Based on Existing Recording Techniques

Low-pass filtering multi-electrode data is a simple, and somewhat idealized, way of asking how functional connectivity may change across timescales. To relate these analyses more closely to existing recording techniques we performed the same analysis using temporal filters based on Ca^2+^, LFP, and fMRI imaging techniques.

**Figure 5:**
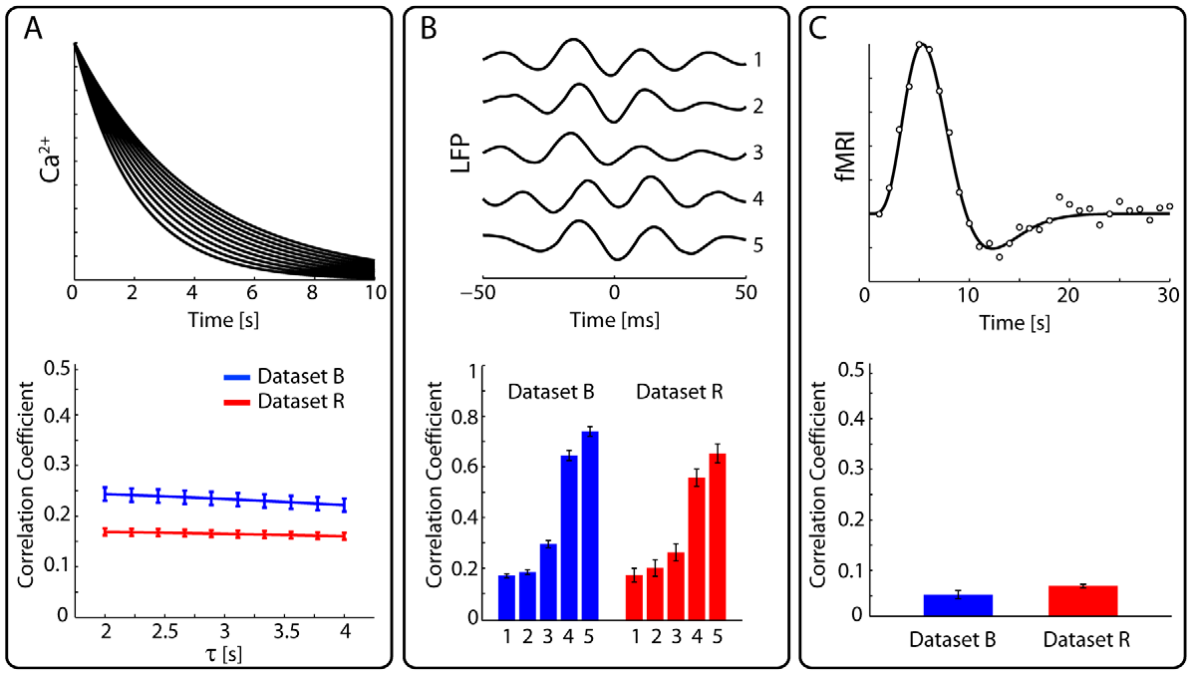
Results from filtering based on existing recording techniques. (A) shows similar results for 10 exponential filters meant to mimic Ca^2+^ imaging. Correlation to fast timescale connectivity was not significantly different over this range of time constants (τ = 2–4 s). (B) shows 5 acausal filters based on data from simultaneous spike-LFP recordings and the correlation between functional connectivity estimated after filtering and fast timescale functional connectivity (sorted for clarity). (C) shows a filter based on a fMRI hemodynamic response function and the resulting correlations. Error-bars denote SEM across segments (N = 5).

To mimic Ca^2+^ imaging we use a typical exponential decay filter [24]–[26]. We simulate 10 filters with time constants ranging from 2–4 s. Functional connectivity estimated after applying these filters was weakly correlated with fast timescale connectivity (R∼0.2), and the correlation did not change significantly over the range of time constants (Fig. 5A). Importantly, this is the connectivity estimated from the raw calcium signals. Novel techniques using deconvolution or statistical inference may allow inferring the spike train directly from the calcium signals [25], [26]. If spikes can be accurately inferred from the imaging signal then fast timescale functional connectivity could be recovered perfectly. Because they are relatively low-pass, simulated calcium signals only allow capturing relatively small parts of the high frequency functional connectivity.

To mimic LFP signals we use acausal filters based on cross-correlation results from primary motor cortex and pre-motor cortex [18]. We used 5 different filters based on 5 different recordings (Fig. 5B). There is large variation in how well the functional connectivity estimated after filtering with these kernels was correlated with fast timescale functional connectivity. This variation persists even when the filters are smoothed to remove any possible edge effects. This may indicate that the precise form of a filter has a strong impact on the quality of the estimated functional connectivity and may deserve further analysis. Still, in ideal cases LFP signals may be about as good as high frequency spike data for the inference of functional connectivity.

Finally, to mimic fMRI signals we use a prototypical hemodynamic response function [20] based on deconvolution results from motor cortex while a human subject performed fixed-frequency finger tapping [27]. We fit this filter using a simple gamma-cosine model [16]. This filter is similar to low-pass filtering at ∼0.2 Hz. We find that correlations with high frequency functional connectivity are rather weak (Fig. 5C). This may suggest that functional connectivity analyses based on fMRI may need to be based on other statistical features of the signal, as in dynamical causal modeling [1]. However, this analysis does not take into account spatial filtering and population effects that may allow more meaningful information about functional connectivity to be extracted from fMRI. These results indicate that even if future imaging techniques could have perfect spatial resolution, without higher temporal resolution recordings, prediction of fast timescale functional connectivity from such signals would be poor.

### Simulations

To examine why functional connectivity might be robust to temporal filtering we perform the above analysis on simulated data generated by a spiking model which captures fast-timescale functional connectivity: a Generalized Linear Model (GLM) [5]–[7]. Whereas Granger causality provides a metric for functional connectivity between continuous signals, the Poisson GLM is an effective method for modeling functional connectivity between point processes (spikes). These two methods make different assumptions about the signals to be modeled and their parameter estimates cannot be interpreted in the same way. However, we can use the GLM as a tool to remove certain characteristics of the original data, such as slow timescale rate modulation, and see the effect of these manipulations on functional connectivity as estimated by Granger causality.

**Figure 6:**
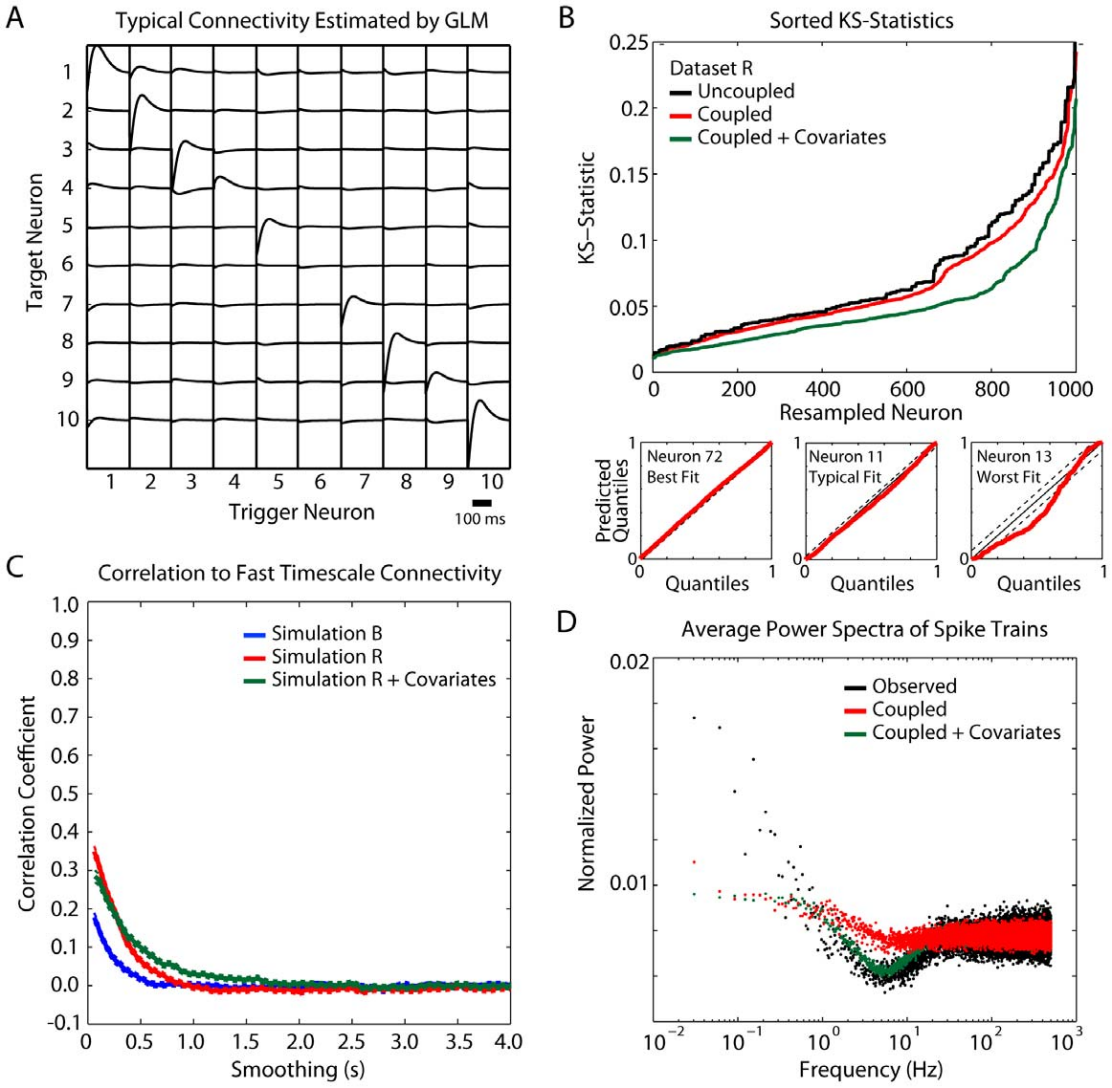
Simulation results using a generalized linear model. (A) shows a typical set of parameters after fitting the spike trains of a subpopulation of 10 neurons. On short timescales (<150 ms), refractory effects dominate spike behavior. However, there are small amplitude interactions between many neurons. (B) shows goodness of fit tests (KS-test on the time-rescaled inter-spike intervals) for three example neurons (bottom) and aggregate KS-statistics for an uncoupled model, a model with coupling, and a model including both coupling and hand velocity (top). Smaller KS-statistics correspond to better fits. After performing the filtering analysis on simulated data, the correlation between connectivity across timescales is robust for all datasets down to ∼5 Hz (C). Error-bars denote SEM across simulations (N = 100). (D) illustrates the differences between the power spectra of the observed and simulated neurons. The model with covariates follows the observed spectra down to ∼1 Hz. However, much of the observed power below 1 Hz is missing from the simulations.

We start with a model that includes post-spike history kernels and coupling terms, both parameterized by raised-cosine basis functions (see Methods for details). These two sets of parameters allow us to model the spiking properties of individual neurons (i.e. refractoriness and burstiness) as well as the functional relationships between pairs of neurons on a certain timescale (100 ms in this case). To make the comparison between real and simulated data as accurate as possible, we fit the parameters of this model to subpopulations of 10 neurons in the original data. The simulated data thus reproduce certain characteristics of the observed spikes, such as firing rates and inter-spike intervals, while removing higher-order correlations as well as any dependence on external variables. Importantly, the GLM attempts to preserve the fast timescale connectivity from the original spike trains (Fig. 6A).

As with previous studies [7], we find that the model accurately fits spike data from motor cortices (Fig. 6B). Using a Kolmogorov-Smirnov (KS) test on the time-rescaled inter-spike intervals to assess goodness-of-fit [28], [29], we find that including coupling terms improves the model's accuracy on cross-validated data. Smaller KS-statistics indicate that the observed and predicted time-rescaled inter-spike intervals are closer together. We then simulate spike trains from the model to generate a proxy dataset with coupling only on timescales <100 ms. The firing rates and inter-spike interval distributions of simulated spikes both match those from the original data. We then perform the analysis above–low-pass filtering, down-sampling, and estimating Granger causality for each timescale (Fig. 6C). If slow timescale connectivity is a direct result of fast timescale connectivity, we expect to observe agreement between fast and slow signal connectivity similar to the original data. However, in these simulations the overall correlations are much lower, and the correlation to fast timescale directed Granger causality drops by ∼50% when the simulated signals are low-pass filtered at 5 Hz.

In some ways this result may not be surprising. In attempting to remove slow-timescale functional connectivity it is likely that we remove important slow-timescale dynamics as well. That is, the simulated spike trains do not show the same rate modulation that real neurons do. For example, the power spectra of the neurons simulated by the coupled GLM match the power spectra of observed spikes trains only for frequencies higher than 10 Hz (Fig. 6D). To address this issue we perform a similar simulation with the addition of an external covariate–in this case, the end-point velocity for the recorded hand trajectories in dataset R. Most of the power in the end-point velocity is between 0.1 and 2 Hz. With the addition of these terms, the simulated neurons match the observed neurons more accurately (Fig. 6B). In this case, the power spectra of simulated neurons match those of the observed spike trains down to 1 Hz, and the correlation between slow timescale and fast timescale connectivity decays more slowly (Fig. 6C and D). However, the robust correlation between functional connectivity on multiple timescales that exists in real neural data is still not observed.

## Discussion

Modeling the interactions between neurons has a number of benefits. Statistical models that incorporate coupling can improve decoding of external variables and give a more complete picture of multi-variate neural signals [5], [30]. Moreover, the estimated connectivity patterns often match the known anatomy and physiology of the brain [3]. In many cases, however, what can be said about connectivity is limited by the spatial and temporal resolution of the recording techniques. It is often assumed that connectivity is the same across all timescales and that connectivity estimated using population signals will reflect connectivity between individual neurons. If methods for estimating functional connectivity are truly capturing synaptic connections, then functional connectivity should be relatively well-preserved across timescales.

On the other hand, there are fundamental limits to how well-preserved functional connectivity estimates will be. As the results from filtering with a hemodynamic response function suggest, low-pass filtering with large smoothing windows removes most of the information about fast time-scale functional connectivity. However, based on the low-pass filtering results it appears that substantial information does exist even when the size of the smoothing window is on the order of a second.

Some previous evidence exists for an agreement between connectivity estimates across timescales. A number of studies have indirectly addressed the issue by using a frequency decomposition of Granger causality [9], [31]–[33]. This method decomposes a single measure of Granger causality into a spectrum. Intuitively, we would expect Granger causality at low frequencies to be preserved by filtering and down-sampling. However, in many cases, it can be hard to interpret this frequency decomposition–for example it can be negative. Closest to our objectives here is a recent study that showed, using simulated local field potentials, that Granger causality is somewhat robust to temporal filtering and down-sampling [34], [35]. However, biological signals may not be as regular as these auto-regressive simulations, and recent evidence suggests that connectivity measures could depend on filter characteristics in a non-trivial way [36]. Here we approached the problem, empirically, by taking real spike signals recorded at high temporal resolution and estimating functional connectivity between filtered versions of the original spike signals. The processed signals are meant to directly mimic the low pass properties of various recorded signals.

In the absence of noise it is, perhaps, not surprising that filtered signals still contain information about fast time-scale connectivity. However, the fact that this connectivity information persists across a coarse segmenting of the data and is robust to additive noise may explain the success of connectivity methods using relatively slow timescale signals, such as fMRI. Agreement between slow timescale functional connectivity and fast timescale functional connectivity appears to hold true across animals, tasks (reaching and sleep), and brain regions (primary motor and pre-motor cortex). The multi-timescale properties of functional connectivity we observed for our data may thus be a general property of neural signals.

Our simulation results further suggest that agreement between slow timescale and fast timescale functional connectivity is not a general property of multivariate time-series and that slow timescale functional connectivity in the brain is not simply caused by fast, pair-wise interactions of the type captured by the GLM. Both stimuli [37] and neural signals [38] themselves operate on multiple timescales. Thus, one possibility is that our simulated signals simply do not capture the multi-timescale structure of real neural signals. LFPs in visual cortex, for instance, have been shown to convey independent information about a stimulus on multiple timescales [22]. From a modeling perspective, the question is how to reproduce the observed correlations between connectivity on different timescales. One option is to extend a GLM-like model to include a wider range of timescales or to include more explicit structure in the relationships between neurons. For instance, it has been suggested that multi-scale interactions could result from non-linear coupling [39] or hierarchical structure [40], [41]. Hierarchical structure is a common characteristic of neuroanatomy [42] as well as behavior [43], and this structure could lead neurons to interact with each other on very slow timescales, in addition to the fast, synaptic-like interactions that the GLM has typically been used to model. Another explanation may be that there is common input driving all of the observed neurons. Since this common input may be multi-timescale itself, this would produce correlations in connectivity across timescales [44].

In the case presented here, adding end-point velocity to the GLM contributed to the correlations at slower timescales. However, this model still does not reproduce the correlations across timescales observed in real data. To fully capture these correlations we may need to include the other sources of common-input. For instance, motor cortex is known to represent planning signals and forces in addition to kinematic variables [45]. Similarly, motor cortex receives broad anatomical connections from the cerebellum and basal ganglia [46]. These broad, slowly varying signals may well shape the low-frequency features of the data that give rise to slow timescale functional connectivity. In sensory systems there is evidence for low frequency noise correlations on many spatial scales [47]–[50], and similar phenomena may occur in motor cortex.

The results presented here suggest that functional connectivity is relatively well conserved across time-scales. However, assuming that neurons or brain areas are interacting on the specific timescales that specific recording techniques observe seems premature. Functional connectivity and correlations between connectivity at different time-scales may depend on a number of different factors such as the regions of the brain we record from, the tasks performed during the recording, and the stationarity of the signals. Moreover, the shape of the filters generating a given signal (i.e. Gaussian, exponential, or hemodynamic) appear to have a substantial influence on how well functional connectivity between neurons can be reconstructed. Having an accurate generative model for the signals may thus be important [51]. Rather than assuming that functional connectivity estimates are direct measures of how the brain works, it seems prudent to interpret connectivity estimates as approximations of an underlying circuit which may be task-dependent and confounded by unobserved factors such as common-input or even activity on timescales outside the range of our recordings.

The results we have presented here are based largely on Granger causality–a linear technique for estimating pair-wise functional connectivity between neurons. Multi-variate approaches or non-linear techniques [52], [53] may yield different estimates of functional connectivity and distinct results for how similar functional connectivity is across timescales. Importantly, non-linear techniques may allow estimation of more complex effects such as coupling across frequencies [39] and gain control or gating [54]. Here we have used Granger causality to provide a base-line for understanding how similar functional connectivity is across timescales.

Finally, it is important to note that there may be large differences between connectivity estimated from recordings of individual neurons and that estimated using populations of neurons. A similar analysis using LFPs may allow for a more direct answer as to whether functional connectivity estimated from slow timescale population signals (such as fMRI) is predictive of fast timescale population signals (see [4], [21], [48]). However, our results suggest that as the *spatial* resolution of imaging techniques increases even functional connectivity estimated from relatively low temporal resolution signals can inform our understanding of how individual neurons interact on fast timescales. Specifically, intermediate temporal resolution recordings (<1 Hz) from individual neurons will be able to provide substantial information about the functional connectivity between those neurons on fast timescales. This finding also suggests that it will be possible to combine connectivity estimates from multiple recording techniques. On the other hand, the basis of this similarity across timescales is not yet clear. Our simulations suggest that slow timescale connectivity is not caused by fast timescale pair-wise interactions, and that the correlations we observe may potentially be explained by the existence of connectivity on many timescales, hierarchical structure, or unobserved common-input. Exploring these types of models promises to shed light on how functional connectivity estimates from different recording techniques relate to one another and on the neural mechanisms that give rise to functional connectivity.

## Materials and Methods

### Ethics Statement

All animal use procedures were approved by the institutional animal care and use committee at the University of Chicago, and conform to the principles outlined in the Guide for the Care and Use of Laboratory Animals (National Institutes of Health publication no. 86-23, revised 1985). Data presented here were previously recorded for use with multiple analyses. Procedures were designed to minimize animal suffering and reduce the number used.

### Recordings

Implantation and recording procedures have been previously described [55]. Briefly, data were collected from two macaque monkeys (*Macaca mulatta*). Data from monkey R were collected during center-out reaching. Data from monkey B were collected across multiple stages of slow-wave sleep (as assessed by visual inspection of local field potentials and eye tracking data). The animals were each implanted with two microelectrode arrays (Blackrock Microsystems, Inc.): one implanted in the primary motor cortex (M1) and one implanted in dorsal premotor cortex (PMd). Each electrode was 1.0 mm in length. The neuronal signals were classified as single- or multi-unit based on action potential shape and inter-spike intervals greater than 1.6 ms. Spike sorting was performed by manual cluster cutting with 143 neurons (78 M1, 65 PMd) discriminated in data from monkey R and 183 neurons (75 M1, 108 PMd) discriminated in data from monkey B. Only well-discriminated single units were used in the subsequent analyses.

### Temporal Filtering Analysis

To examine the effects of temporal filtering and noise on the estimation of connectivity each spike train was low-pass filtered (through convolution with a Gaussian) and down-sampled proportional to this smoothing (factor of 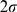). We then estimate the Granger causality [23] between all pairs of signals, in some cases after adding uncorrelated fixed-SNR Gaussian noise to each signal. We chose Granger causality due to its popularity in analyzing LFP, EEG, and fMRI data and its relative simplicity. For two signals 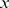 and 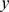 the pair-wise directed Granger causality (equation 1) is the log-likelihood ratio between the univariate and bivariate auto-regressive models of order k:
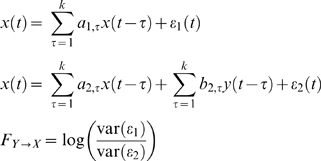
(1)


Low-pass filtering and down-sampling may not have completely independent effects on the correlations across time-scales. However, combining the two is meant to mimic physical recording techniques where how fast we sample is often determined by how fast the underlying signal varies.

To prevent any biases from over-fitting we estimate the parameters of the auto-regressive model and the Granger causality 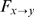 on different segments of the data. The correlations between Granger causality estimates were then also computed on different segments of the data. For practical reasons, the fastest timescale we study is 60 ms (16.67 Hz). Since spikes are sparse signals, using higher resolution data requires large model orders to produce non-zero Granger causality. Moreover, as the model order increases so does the potential for over-fitting. Here we use k = 8. Higher model orders do no significantly improve the cross-validated fraction of variance explained, and we find that, generally, the model order (over the range k = 4 to k = 10) does not have a significant impact on the correlation results presented here.

### Simulations

The generalized linear model [2], [5]–[7] assumes that spikes are generated by a doubly stochastic Poisson process (Cox process). The conditional intensity (instantaneous firing rate) of each neuron depends on a short history of the activity from all neurons. Given a history 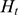 of the activity of 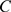 neurons and model parameters 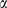, the conditional intensity for neuron 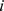 is given by equation 2 and spikes are drawn from a Poisson distribution with this rate.
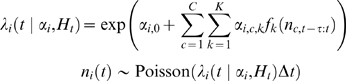
(2)


Where 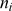 denotes the number of spikes fired by neuron 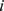 in a short time window. We fit the model parameters 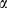 using maximum likelihood estimation and 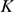 raised cosine basis functions 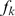, similar to [5]. In (2) 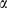 parameterizes both post-spike filters and coupling filters. Finally, to incorporate end-point velocity we use a variation of the model used by [7].
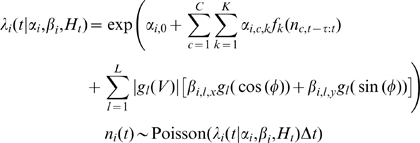
(3)


Where 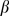 parameterizes the dependence of each neuron's firing rate on hand-direction, and we again use raised-cosine basis functions 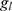 to expand the covariate in time.

To assess goodness-of-fit we can use the time-rescaling theorem and perform a Kolmogorov-Smirnov test to compare the rescaled inter-spike intervals with those predicted by the GLM [28], [29]. After fitting each of these models to the original spike data, we simulate spikes at high temporal resolution (1 ms). We then follow the methods above to see how robust the connectivity of these simulated systems is to temporal filtering.
